# Clinicopathological and Prognostic Significance of Survivin Expression in Patients with Oral Squamous Cell Carcinoma: Evidence from a Meta-Analysis

**DOI:** 10.1371/journal.pone.0116517

**Published:** 2015-02-24

**Authors:** Shang Xie, Hui Xu, Xiaofeng Shan, Baozhong Liu, Kan Wang, Zhigang Cai

**Affiliations:** Department of Oral and Maxillofacial Surgery, Peking University School and hospital of Stomatology, Beijing 100081, China; Duke Cancer Institute, UNITED STATES

## Abstract

**Background:**

Survivin has been proposed as a promising prognostic marker in oral squamous cell carcinoma (OSCC), but the published data on survivin expression in patients with this condition are controversial. To address this we performed a meta-analysis systematically to assess the clinicopathological and prognostic significance of survivin expression in OSCC.

**Methods:**

We searched PubMed, Embase, Web of Science and Ovid databases for papers investigating the clinicopathological and prognostic significance of survivin expression in OSCC. The pooled odds ratios (ORs) and corresponding 95% confidence intervals (CIs) were used to determine the relevance of survivin.

**Results:**

A total of 15 papers, including 1040 cases in which survivin expression was detected by immunohistochemistry (IHC) or reverse transcription polymerase chain reaction (RT-PCR), were included. A meta-analysis of clinicopathological variables revealed a correlation between survivin expression and lymph node metastasis (OR = 0.62, 95% CI = 0.44–0.88, p < 0.05) and clinical stage (OR = 0.63, 95% CI = 0.41–0.96, p < 0.05). However, no significant associations were found between survivin expression and tumor differentiation grade (OR = 0.72, 95%CI = 0.26–1.11, p > 0.05), depth of invasion (OR = 0.76, 95% CI = 0.50–1.14, p > 0.05), age (OR = 0.78, 95% CI = 0.48–1.29, p > 0.05) or gender (OR = 1.31, 95% CI = 0.86–2.01, p > 0.05). Subgroup analysis using stratified detection methods showed no significant associations between the expression of survivin protein and clinicopathological variables in OSCC. A correlation between survivin expression and poor prognosis of patients with OSCC (HR = 1.62, 95% CI = 1.23–2.01, p < 0.05) was demonstrated.

**Conclusion:**

Survivin is a potential prognostic marker of OSCC. Future studies with larger sample sizes and well-designed inclusion criteria will be needed to dissect the role of survivin expression in determining the clinicopathological features and/or prognosis of OSCC.

## Introduction

Oral cancer is one of the 10 most common cancers in the world. Its high mortality rate and the disfigurement that survivors may suffer gives rise to a considerable global public health burden [1,2]. About 90% of malignant oral neoplasms are oral squamous cell carcinomas (OSCC), followed by adenocarcinoma and, rarely, other types of tumor [3]. Despite advances in treatment for OSCC, the 5-year survival rate remains poor [4–7] and further studies of risk factors and optimal means for early diagnosis of OSCC are imperative.

Widely used and conventional prognostic factors, such as the presence of lymph node metastases, clinical stage and tumor size, are not always of use in the early diagnosis of OSCC. Further, the inherent heterogeneity of both tumor biology and patient factors between affected individuals means that reliable prognostic factors for OSCC are currently lacking. Additional factors, including molecular markers, are likely to be needed to explain the mechanism of development and occurrence of OSCC.

Biomarkers are important in establishing an accurate diagnosis and also can provide prognostic data. For instance, alpha fetoprotein (AFP) is widely used as one facet in the process of diagnosing liver cancer. However, to date, no specific tumor marker has been identified for use in OSCC.

In the 1990’s, survivin, a member of the ‘inhibitor of apoptosis’ (IAP) family, was found to be over-expressed in tumors and fetal tissues, but not in normal adult tissues [8]. Functionally, survivin has been demonstrated to inhibit caspase-3 and -7 activity, suggesting a mechanism by which it may suppress apoptosis [9]. Li et al. have also shown survivin specific expression in the G2/M phase of the cell cycle suggesting a role in the regulation of cell division, overcoming the apoptotic checkpoint and causing the default induction of apoptosis [10]. In addition, survivin expression has been demonstrated to be important in the process of angiogenesis [11]. A report by Pennati and colleagues highlights the impact of the regulation of survivin gene expression on key potential survivin functions, including reduced tumor growth potential and increased apoptotic rate [12]. Together, these studies provide a basis for the consideration of survivin as a potential marker of tumors.

This has been directly addressed in several cancer types with regard to determining prognosis [13–15], but its prognostic value in OSCC remains controversial. Some studies have shown that survivin expression might be an indicator of OSCC [16–18], though this has not been universally replicated [19,20]. In many cases, these studies are limited by small sample size with their inconsistent results being potentially attributable to random error. We have therefore undertaken a meta-analysis to systematically assess the role of survivin as clinicopathological and prognostic molecular marker of OSCC.

## Materials and Methods

### Search strategy

We searched PubMed, Web of Science, Ovid and Embase databases on April 3rd 2014 for articles in English that met the following search criteria: (1) survivin OR BIRC5 OR baculoviral inhibitor of apoptosis repeat-containing 5, and (2) oral OR mouth OR lip OR buccal OR tongue OR gingiva OR palate OR mouth floor, and (3) tumor OR cancer OR carcinoma OR neoplasm. Retrieved papers were independently screened by two authors according to the title, abstract and type of article, and irrelevant papers were dropped out. A manual review of the references cited in the included articles was also performed to highlight articles that might have been missed by the original search strategy. All relevant papers were then assessed using inclusion and exclusion criteria for meta-analysis, as described below.

### Inclusion/exclusion criteria

The following criteria were set and reviewed by two independent authors: (1) survivin expression of OSCC was evaluated by immunohistochemistry (IHC) or RT-PCR analysis; (2) the levels of survivin expression were used to estimate the relation between survivin and clinicopathological variables and/or prognosis of OSCC; (3) articles were published in English as full paper; (4) odds ratios (ORs) for estimating clinicopathological variables were provided or were extractable from the original articles; sufficient information was provided in relation to survivin expression and prognosis of OSCC; (5) when multiple publications from a particular research group reported data from overlapping samples, the study reporting the largest dataset was included; (6) reviews, meta-analyses, letters, conference abstracts, case reports and non-English language articles were excluded; (7) articles that provided no sufficient information on prognosis or ORs were excluded.

### Data extraction

All data were independently reviewed and extracted by two authors (Xie and Xu). Differences between reviewers were resolved by discussion and through consultation, if necessary. The following characteristics were collected from each study: first author, publication year, subjects’ ethnicity, country, sample size, cut-off values, survivin subcellular location, clinicopathological and prognostic variables, and other relevant data.

### Quality assessment

We used the Newcastle-Ottawa Scale (NOS) [21,22] to evaluate the methodological quality of all included studies. The NOS system categorizes studies using three dimensions (selection of cohort, comparability of cohort, and ascertainment of outcome) with each dimension being assessed by eight items. A star system is used to assess the quality of all included studies. All of the included studies were awarded a maximum of four stars in selection, two stars in comparability, and three stars in exposure. The NOS ranges from zero to nine stars, with more stars indicating a better quality. The assessment was performed independently by two authors and any discrepancy was resolved by discussion.

### Statistical analysis

Data management and analysis were performed with STATA 11.0 software (Stata Co., College Station, TX). The ORs with corresponding 95% CIs were used to define the association between survivin expression and clinicopathological variables in patients with OSCC. We extracted the clinicopathological variables and combined them by meta-analyses. The clinicopathological variables included: the presence or absence of lymph node metastasis; clinical stage I and stage II versus stage III and stage IV; good differentiation versus moderate-poor differentiation; depth of invasion T1 and T2 versus T3 and T4; male versus female; and age less than 60 versus more than 60 years.

For prognostic parameters, the hazard ratios (HRs) with corresponding 95% CIs were calculated to estimate the effect of survivin expression on survival rates. If the authors reported HR and 95% CI, data were directly extracted from the original articles. Otherwise, these data were calculated by the methods described by Parmar et al. [23] and Tierney et al. [24]. Kaplan-Meier survival curves were read by Engauge Digitizer version 4.1.

In order to calculate the heterogeneity of the studies we included, the Chi-Square test was used and significance was set at p < 0.05 [25]. The inconsistency index I^2^ was calculated to assess the variation caused by heterogeneity. Where p > 0.10 and I^2^ < 25%, the fixed-effect model was used, which assumes the same homogeneity of effect size across all studies. Where p < 0.10 and I^2^ > 25%, inter-study heterogeneity was deemed statistically significant, and a random effects model was employed. Funnel plots were used to detect underlying publication bias, with the plots’ asymmetry being estimated by Begger and Egger’s linear regression [26]. Sensitivity analysis was performed to identify the influence of the individual studies on the combined OR. In this analysis, we excluded each study individually to assess its influence on the results.

### Trial sequential analysis (TSA)

According to the Cochrane Handbook, meta-analyses and systematic reviews are considered to be the best available evidence if all eligible trials are included. However, ‘the best available evidence’ might not be equal to ‘sufficient evidence’. Based on this issue, we applied the TSA to estimate the robustness of the current conclusions [27,28]. In our article, we planned to calculate the required power to collect adequate information and evaluate how many subjects would be necessary to make these robust conclusions. The required power was based on the assumption of a plausible relative risk of 10% with low risk bias, and we adopted the risks for a type I error (α) of 5%, a type II error (β) of 20%[27]. According to the required power and risk for type I and type II errors, TSA monitoring boundaries were built. If a TSA monitoring boundary is crossed with Z-curve before the required power is reached, further trials are unnecessary. Otherwise, it is necessary to continue performing trials.

## Results

### Study selection and characteristics of included studies

**Fig 1:**
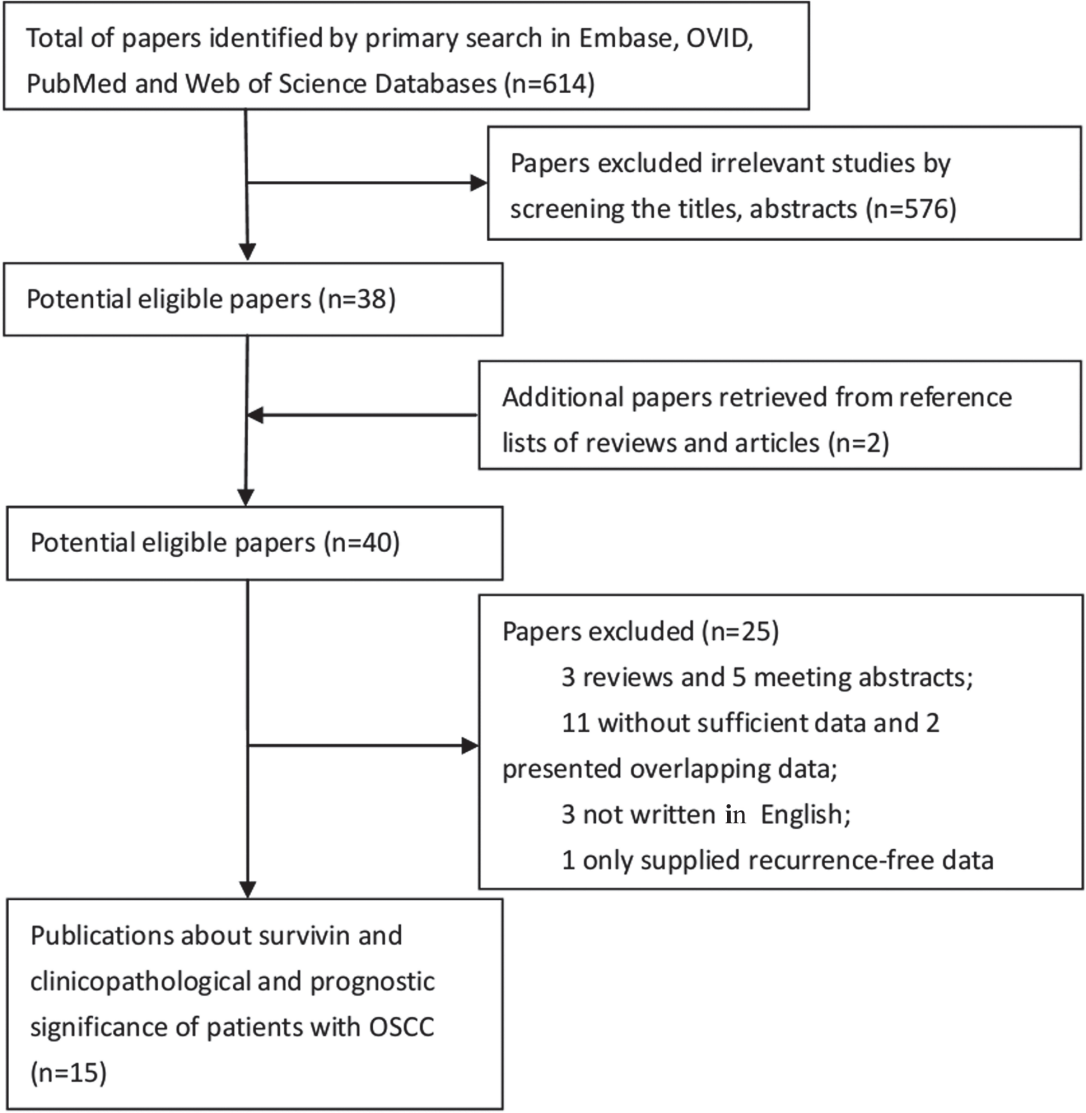
Flow diagram of the literature search process.

A total of 614 articles were retrieved by the database search, of which 576 studies were excluded as being irrelevant to OSCC or survivin, or due to lack of clinicopathological or prognostic data. A further two potentially eligible papers were obtained by screening the references of reviews. After more detailed evaluations of the 40 potentially eligible articles, three were excluded as being reviews. A further two studies were excluded due to the presence of overlapping data, and 11 more failed to provide sufficient data to warrant inclusion. Five studies were meeting abstracts only, and were therefore also excluded. Three were unavailable in English. A single further paper was excluded because it provided only recurrence-free data without any discussion of clinicopathological features or survival rates. Fifteen papers were therefore included in the meta-analysis to estimate the clinicopathological and prognostic significance of survivin as a potential marker in OSCC [16–20,29–38]. The search process is shown in Fig. 1.

The key information extracted from each eligible paper is summarized in Table 1 (Details were shown in S1 Table), including the first author, publication year, country of origin, number of cases, clinicopathological and prognostic variables and other necessary data. Of the 15 studies included in this meta-analysis, eight studies investigated the association between survivin expression and clinicopathological parameters, two papers studied its prognostic significance in patients with OSCC, and five papers investigated both of these aspects. The number of cases included ranged from 13 to 251, and a total of 1040 cases were included in the meta-analysis. Data were unfortunately incomplete regarding the subcellular location of survivin expression: of the 15 studies, three gave no information on subcellular location, while four described a cytoplasmic location, and the remainder described both cytoplasmic and nuclear survivin.

**Table 1:** Clinicopathological and methodological features of eligible studies.

First Author	Year	Country	Cases	AT^#^	NOS*	C. Features**	Prognosis	Method	Cut-off value	HR Estimate (95% CI)	Location
L Lo Muzio	2003	Italy	110	NA	8	N,S,D,A,G	HR	IHC	<5%	HR = 2.28 (1.49–4.04)	cytoplasm and/or nucleus
C Tanaka	2003	Japan	71	NA	8	N,S,T,D,G,A	NA	IHC	IHC score<100	NA	cytoplasm
M-J Kim	2005	South Korea	113	NA	7	NA	sur. curve	RT-PCR	median value	HR = 2.78 (1.53–5.08)	---
C-Y Lin	2005	Taiwan	96	NA	7	N,S,T,D,G	sur. curve	IHC	<25%	HR = 1.94 (1.11–3.37)	Cytoplasm
G Marioni	2005	Italy	18	NA	7	N,T,D,G,A	NA	IHC	<9.5%	NA	nucleus and/or cytoplasm
C Jane	2006	India	38	NA	7	D	NA	IHC	<5%	NA	cytoplasm
K Freier	2007	Germany	251	AR/AC	7	T,N	sur. curve	IHC	<20% cytoplasm and <10% nucleus	HR = 1.39 (0.94–2.07)	nucleus and/or cytoplasm
S De Maria	2009	Italy	22	NA	8	N,S,D,A,G	NA	IHC	<30%	NA	nucleus and/or cytoplasm
Z Khan	2009	India	29	NA	7	N,S,T,D	NA	IHC	<10%	NA	nucleus and/or cytoplasm
Y-H Kim	2010	South Korea	38	NA	7	N,D,S,G,A	sur. curve	IHC	<20%	HR = 1.87 (0.56–6.25)	nucleus and/or cytoplasm
G Lodi	2010	Italy	17	NA	7	G,A	NA	Real-time RT-PCR	median value	NA	---
L-P Su	2010	China	68	No	7	N,S,T,D,G,A	HR	RT-PCR	median value	HR = 2.71 (1.46–5.10)	---
S-X Li	2012	China	13	NA	8	G,A	NA	ISH	>0%	NA	nucleus and/or cytoplasm
M-B Zhang	2013	China	110	NA	8	NA	sur. curve	IHC	mean value	HR = 1.12 (0.54–2.30)	cytoplasm
M Dogan	2014	Turkey	46	NA	8	N,D,G	NA	IHC	>0%	NA	nucleus and/or cytoplasm

^#^AT: Adjuvant therapy

*NOS: Newcastle-Ottawa Scale

**C. Features: clinicopathological features; AR: adjuvant radiotherapy; AC: adjuvant chemotherapy; NA: not available; sur. curve: survival curve; IHC: immunohistochemistry; HR: hazard ratio; N: lymph node; S: clinical stage; T: depth of invasion; D: differentiation; G: gender; A: age

### The results of quality assessment

Using the Newcastle-Ottawa Scale system, quality assessment for the included studies yielded either seven stars or eight stars (shown in Table 1), indicating that all were of moderate to high quality.

### Survivin expression and clinicopathological parameters in patients with OSCC

We studied the relationship between high survivin expression and clinicopathological features in patients with OSCC. To identify an appropriate statistic model for combining the data, we performed heterogeneity analyses for all clinicopathological variables, including models for lymphatic metastasis, clinical stage, depth of invasion, cell differentiation, gender, and age. The results are shown in Table 2. The I^2^ ranges from 0.0% to 19.3%, and p value ranges from 0.266 to 0.832, indicating a lack of significant inter-study heterogeneity. The fixed-effect model was therefore used to combine the pooled ORs and CIs, suggesting a significant relationship between survivin expression and lymphatic metastasis (OR = 0.62, 95% CI = 0.44–0.88, p < 0.05) and clinical stage (OR = 0.63, 95% CI = 0.41–0.96, p < 0.05). However, no significant associations were found between survivin expression and cell differentiation, depth of invasion, gender or age (Table 2).

**Table 2:** Meta-analyses estimating the relevance of survivin with regard to clinicopathological variables.

Clinicopathological variables	No. of studies	Cases	Pooled data		Test for heterogeneity		
			OR (95% CI)	p	Chi^2^	p	I^2^
***Gender (male / female)***							
*All studies*	10	499 (311/188)	1.31 (0.86–2.01)	0.21	9.51	0.392	5.30%
*Subgroup*							
Protein expression (IHC)	7	401 (270/131)	1.19 (0.73–1.94)	0.493	7.56	0.272	20.6%
mRNA expression (PCR/ISH)	3	98 (41/57)	1.83 (0.75–4.46)	0.181	1.63	0.443	0.0%
***Age (< 60 yr/ >60 yr)***							
*All studies*	8	357 (134/223)	0.78 (0.48–1.29)	0.337	4.47	0.724	0.00%
*Subgroup*							
Protein expression (IHC)	5	234 (79/155)	0.77 (0.41–1.43)	0.400	4.47	0.346	10.5%
mRNA expression (PCR/ISH)	3	123 (55/68)	0.82 (0.36–1.86)	0.631	0.02	0.988	0.0%
***UICC stage (I+II / III+IV)***							
*All studies*	7	434 (206/228)	0.63 (0.41–0.96)	0.033	6.83	0.337	12.10%
*Subgroup*							
Protein expression (IHC)	6	366 170/196)	0.77 (0.47–1.24)	0.281	3.54	0.617	0.0%
mRNA expression (PCR/ISH)	1	68 (36/32)	0.26 (0.10–0.72)	0.010	0.00	---	---
***Depth of invasion (T1+T2 / T3+T4)***							
*All studies*	6	533 (296/237)	0.76 (0.50–1.14)	0.19	2.12	0.832	0.00%
*Subgroup*							
Protein expression (IHC)	5	465 (257/208)	0.74 (0.47–1.17)	0.200	2.08	0.720	0.0%
mRNA expression (PCR/ISH)	1	68 (39/29)	0.83 (0.32–2.17)	0.701	0.00	---	---
***Differentiation (well / moderate + poor)***							
*All studies*	10	536 (271/265)	0.72 (0.46–1.11)	0.14	5.89	0.751	0.00%
*Subgroup*							
Protein expression (IHC)	9	468 (250/218)	0.83 (0.51–1.35)	0.449	3.91	0.865	0.0%
mRNA expression (PCR/ISH)	1	68 (21/47)	0.35 (0.12–1.06)	0.064	0.00	---	---
***Lymph node (without / with metastasis)***							
*All studies*	10	749 (354/395)	0.62 (0.44–0.88)	0.008	11.15	0.266	19.30%
*Subgroup*							
Protein expression (IHC)	9	681 (316/365)	0.70 (0.48–1.02)	0.062	7.81	0.453	0.0%
mRNA expression (PCR/ISH)	1	68 (38/30)	0.27 (0.10–0.73)	0.010	0.00	---	---

Since the studies included in the meta-analysis assessed survivin expression at both the transcriptional and the protein level, we undertook a subgroup analysis to assess the correlation between survivin expression and the clinicopathological features of OSCC using both detection methods. Lymph node metastasis and the clinical stage of OSCC were correlated with survivin expression at the genetic level, though no significant relationship between clinicopathological features and survivin overexpression at the protein level was identified (Table 2).

**Fig 2:**
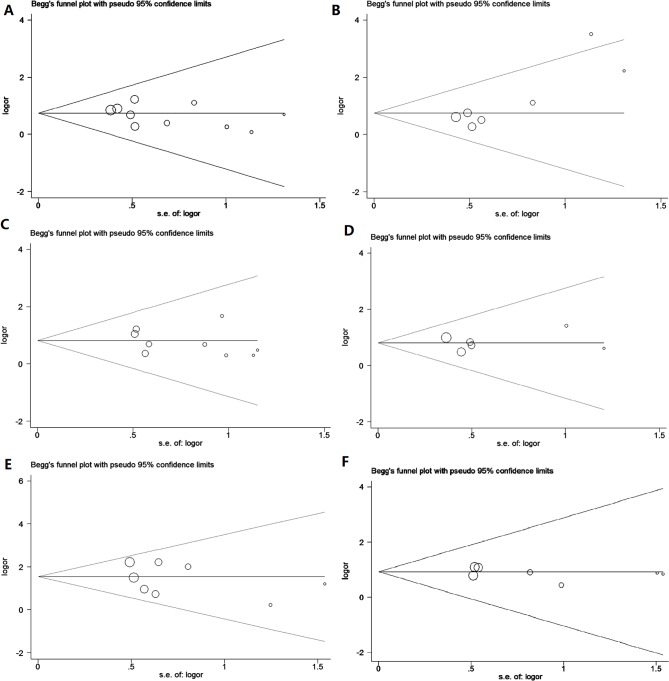
Funnel plots assessing possible publication bias for clinicopathological features (A: lymph node involvement; B: clinical stage; C: cell differentiation; D: depth of invasion; E: gender; F: age).

**Fig 3:**
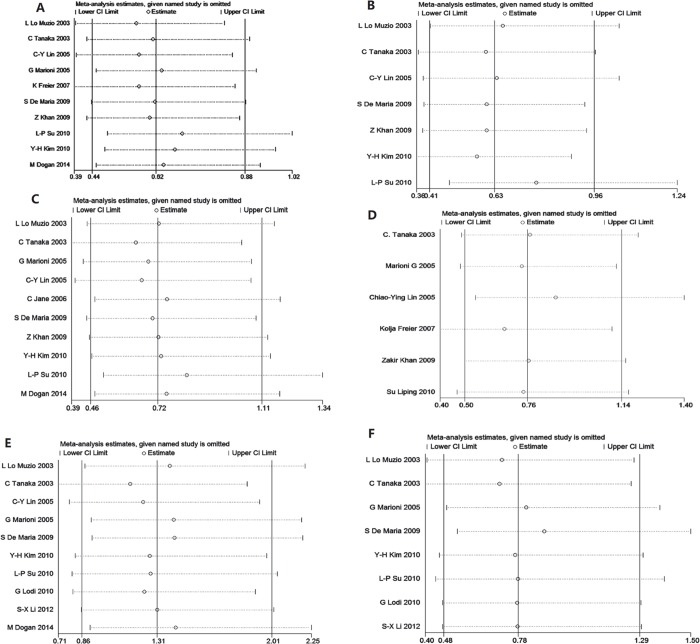
Sensitivity analysis for clinicopathological features (A: lymph node involvement; B: clinical stage; C: cell differentiation; D: depth of invasion; E: gender; F: age).

Funnel plots were used to evaluate possible publication bias, and are shown in Fig. 2. These were symmetrical for all clinicopathological variables models indicating no obvious publication biases. In order to estimate the stability of the results, the ‘leave-one-out’ sensitivity analysis was performed for each study. Fig. 3 demonstrates that the OR estimates changed only the between the lower and upper CI limits for the pooled data, suggesting that none of the included articles had a disproportionate effect on the overall results.

### Impact of survivin expression on survival rates of patients in OSCC

**Fig 4:**
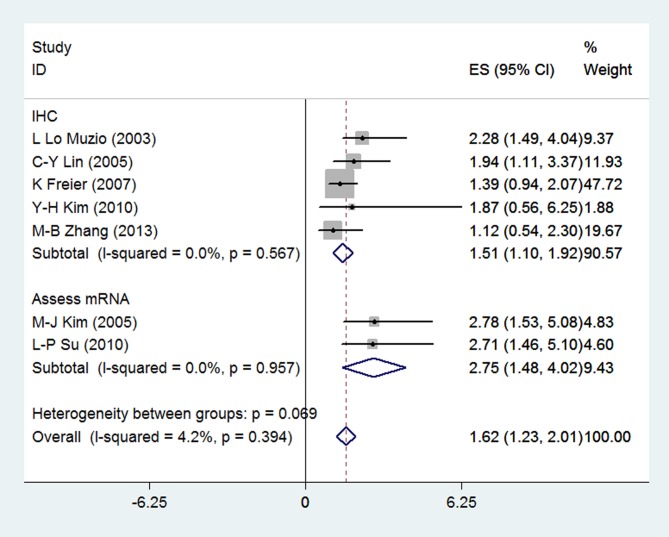
Forest plot estimating prognosis of patients with OSCC.

The meta-analysis included seven assessments of the potential correlation between survivin expression and survival rates, including 786 patients. There was no significant heterogeneity among these articles (I^2^ = 4.2%, p_(Q-test)_ = 0.394), and a fixed-effect model was used to combine the HR and 95% CI. The overall HR was 1.62 (95% CI = 1.23–2.01, p < 0.05), indicating that survivin overexpression is correlated with prognosis in OSCC. To further explore the associations between survivin gene or protein expression and prognosis, we carried out a subgroup analysis stratified by these two detection methods. The results suggested that the pooled HR of survivin mRNA and protein expression were 2.75 (95% CI = 1.48–4.02; I^2^ = 0.0%; p_(Q-test)_ = 0.957; p <0.05) and 1.51 (95% CI = 1.10–1.92; I^2^ = 0.0%; p_(Q-test)_ = 0.567; p <0.05), respectively, indicating that both survivin gene expression and protein expression were significantly correlated with prognosis in patients with OSCC (Fig. 4, S2 Table).

**Fig 5:**
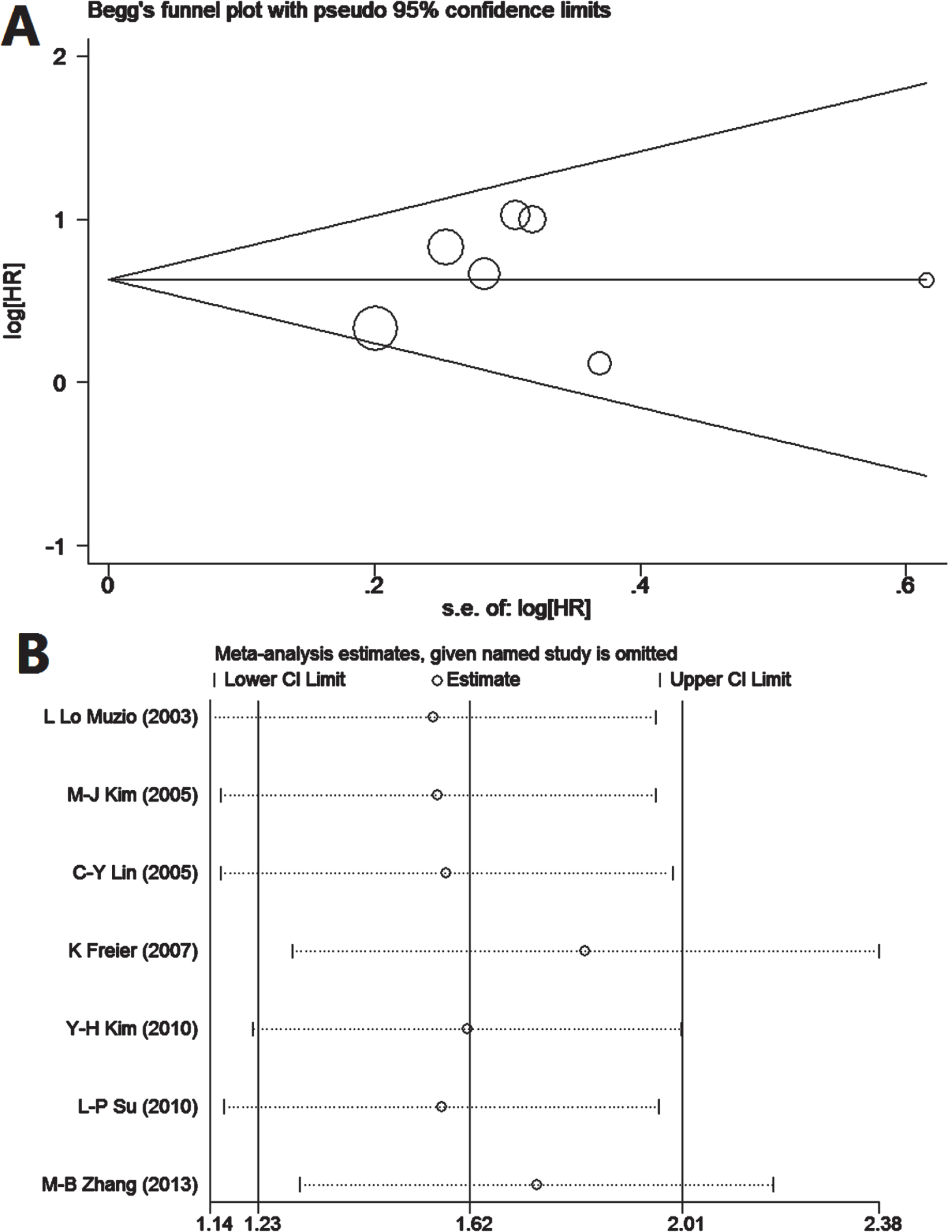
Funnel plot and sensitivity analysis for prognosis of patients with OSCC (A: Funnel plot; B: sensitivity analysis).

Potential publication bias and sensitivity were assessed using Begg’s funnel plot and sensitivity analysis (Fig. 5). Begg’s Test (p = 1.000) and Egger’s Test (p = 0.663) demonstrate no obvious publication bias in this meta-analysis of prognosis in OSCC. The sensitivity analysis produced variation only between the CI limits, indicating that the results from our meta-analysis are stable and credible.

### Trial sequential analysis (TSA)

**Fig 6:**
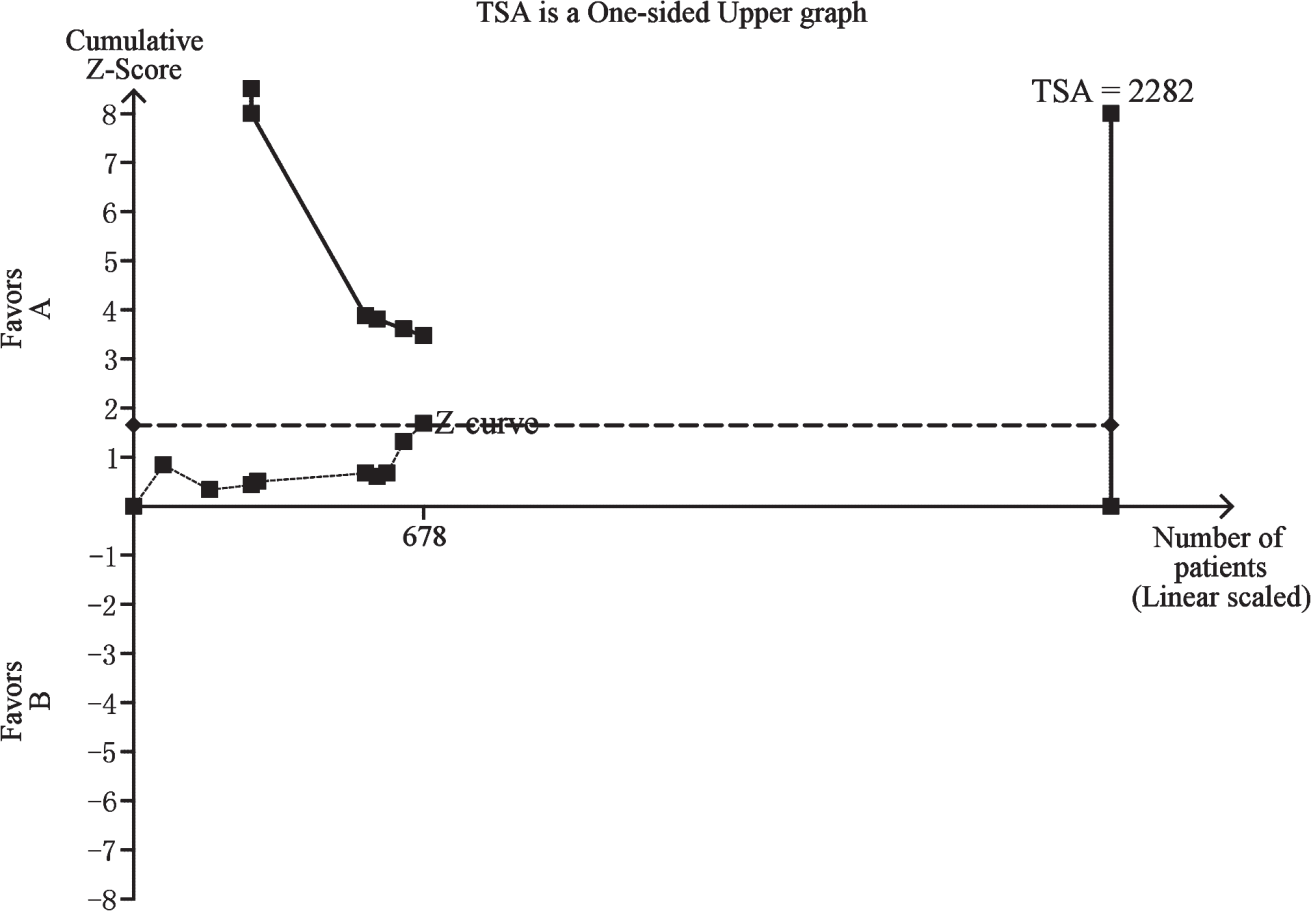
The information size to demonstrate the relevance of survivin protein expression with lymph node metastasis. The dashed curve represents the cumulative Z-curve. The solid line represents the trial sequential monitoring boundary.

Fifteen trials (1040 subjects) were used to investigate the relevance of survivin expression with clinicopathological variables and OSCC prognosis. Using the relevance of survivin protein expression with lymph node metastasis of OSCC (including 9 trials with 678 patients) as an example, we performed the TSA and found that the required power to demonstrate clear conclusions was 2282 subjects (Fig. 6). As Fig. 6 shows, the cumulative z-curve does not cross the trial monitoring boundary before reaching the required information size, which indicates that the cumulative evidence is insufficient and further trials are necessary. The results of other groups were not shown as the study methods were similar.

## Discussion

In 1997, Ambrosini and his colleagues discovered survivin by hybridization screening of the human P1 genomic library with the effector cell protease receptor-1 cDNA [8]. The gene encoding survivin, located on chromosome 17q25, encodes a structurally unique member of the inhibitor of apoptosis protein (IAP) family that contains a single conserved BIRC5 domain (baculoviral inhibitor of apoptosis repeat-containing 5) but no RING finger [8,39]. Abundant expression of survivin has been found in a variety of malignancies, including lung, liver, breast and gastric cancer [40–43], but is not detected in normal adult tissues. This suggests that survivin expression may play a role in tumorigenesis, and has led to its being proposed as a promising molecular marker in the diagnosis and prognostication of malignancy.

Several studies have aimed to assess the potential association between survivin expression and the presentation and prognosis of cancers. Where results were unclear or controversial, meta-analyses have been performed looking at survivin’s role in lung cancer [44], colorectal cancer [45], bladder cancer [46], esophageal cancer [47], and gastric cancer [48], among others. Here we report a similar meta-analysis of the role of survivin as a prognostic indicator in OSCC.

In this meta-analysis, we included 15 eligible articles comparing the different clinicopathological features or survival rates of patients with OSCC according to survivin expression. Our results suggested that expression of survivin at both the transcript and protein level was correlated with prognosis in patients with OSCC, indicating that survivin may be a promising molecular marker in this disease.

We further demonstrated that survivin mRNA expression is correlated with the presence of lymph node metastasis and with clinical stage, but does not appear to be related to other clinicopathological features. We failed to find any correlation between these clinicopathological features and survivin protein expression These apparently conflicting data for survivin transcript and protein expression might be explained, at least in part, by the existence of splice variants [29,49,50], since differently spliced forms of the human survivin transcript have been suggested to perform different functions in distinct subcellular compartments [51,52].

All the results of heterogeneity analyses, as well as sensitivity analysis and assessment of publication bias support the results from this meta-analysis as robust and credible. Nevertheless, it is important to take into account some limitations of our study.

First, articles were limited to those published in English, which will necessarily exclude potentially relevant studies in other languages. The tendency to publish positive findings over negative results may also introduce some bias. Second, the methods of generating HR estimates and 95% CI varied between studies: there were only two studies which offered these parameters directly, and the extraction of data from the survival curves in other five studies may give rise to inconsistencies. Third, the roles of survivin may vary with its subcellular location [53]. In this meta-analysis, several studies did not differentiate between nuclear and cytoplasmic survivin expression which may impact on the utility of the data they provide. Fourth, all the included articles were retrospective studies; ideally, prospective studies would be required to generate more robust conclusions.

Despite these limitations, by combining the data from different studies our meta-analysis permits the conclusion that there are significant associations between survivin overexpression and survival rates in patients with OSCC, indicating that survivin may be a potential biomarker for OSCC. Future studies with larger sample sizes and well-designed inclusion criteria will be needed to dissect the role of survivin expression in determining the clinicopathological features and/or prognosis of OSCC.

## Supporting Information

S1 PRISMA Checklist PRISMA checklist. (DOC)

S1 Table Original data for clinicopathological and prognostic characteristics of included studies. (XLSX)

S2 Table Statistical results for the association between survivin expression and prognosis of OSCC. (XLSX)
